# Vascular norepinephrine responsiveness index for early risk stratification in emergency department septic shock: a real-world cohort study from a Latin American sepsis code registry

**DOI:** 10.3389/fmed.2026.1941982

**Published:** 2026-09-30

**Authors:** Miguel Santiago Paiba-Montaña, Juan David Bustos Acosta, Liliana Patricia Acero Galvis, Jonathan Alexander Guezguan Pérez, Yardany Rafael Méndez Fandiño

**Affiliations:** 1 Universidad Pedagógica y Tecnológica de Colombia, Tunja, Boyacá, Colombia; 2 Hospital Regional de Duitama, Duitama, Boyacá, Colombia

**Keywords:** mortality, norepinephrine, real-world evidence, septic shock, vascular responsiveness

## Abstract

**Background:**

Septic shock is characterized by heterogeneous vascular responsiveness to norepinephrine. The vascular norepinephrine responsiveness index (VNERi), calculated as diastolic arterial pressure divided by the product of norepinephrine dose and heart rate, has been proposed as a bedside risk-stratification marker. Its performance in routine emergency department care remains uncertain.

**Methods:**

We conducted a single-center retrospective cohort study of adults with septic shock treated with norepinephrine in the emergency department from 2023 to 2025. The index time was the first protocolized invasive hemodynamic assessment after norepinephrine initiation. The primary outcome was 28-day in-hospital mortality. Logistic regression evaluated the association between VNERi and mortality, adjusting for age, Sequential Organ Failure Assessment score, Charlson comorbidity index, and serum lactate. Discrimination was compared with that of the mean arterial pressure-to-norepinephrine dose ratio (MAP/NE) using paired receiver operating characteristic curves. Non-linearity was assessed using restricted cubic splines. The previously reported value of 6.7 and a cohort-specific cutoff derived using the Youden index were explored.

**Results:**

Among 238 patients, 70 experienced 28-day in-hospital mortality. Median age was 72 years, 52.9% were female, median Charlson comorbidity index was 4, and mean Sequential Organ Failure Assessment score was 7.4. Each 1-unit increase in VNERi was independently associated with lower odds of mortality (adjusted odds ratio, 0.85; 95% CI, 0.79–0.91). VNERi showed modestly greater discrimination than MAP/NE (AUROC, 0.737 [95% CI, 0.675–0.799] vs. 0.667 [95% CI, 0.592–0.742]; *p* = 0.012). The spline model fit better than the linear specification (*p* = 0.014). Mortality was 48.0% with VNERi <6.7 and 8.8% with VNERi ≥6.7. The cohort-specific cutoff of 7.6 provided only a marginally higher Youden index than 6.7 (0.475 vs. 0.470).

**Conclusions:**

Lower VNERi was independently associated with higher 28-day in-hospital mortality and showed modestly greater discrimination than MAP/NE. Prospective multicenter validation using standardized measurement timing and external validation of candidate thresholds are required before clinical implementation.

## Introduction

Septic shock is a subset of sepsis in which profound circulatory, cellular, and metabolic abnormalities are associated with a greater risk of mortality (1). The global burden of sepsis remains substantial, with important implications for emergency and critical care systems worldwide (2). Despite standardized definitions, septic shock is not hemodynamically uniform. Vasoplegia, relative hypovolemia, myocardial dysfunction, endothelial injury, and microcirculatory abnormalities may coexist in different proportions, leading to heterogeneous responses to vasopressor therapy (3–6).

Norepinephrine (NE) remains the first-line vasopressor for septic shock. Contemporary guidelines support an initial mean arterial pressure (MAP) target of 65 mmHg, but achieving this target does not necessarily indicate equivalent vascular responsiveness across patients (4, 7, 8). A given MAP may require markedly different NE doses, whereas NE dose alone does not account for the arterial pressure achieved (9, 10). This limitation is particularly relevant in emergency departments, where early decisions often rely on routinely available bedside variables.

Diastolic arterial pressure (DAP) may approximate arterial tone in vasodilatory shock, especially when interpreted together with heart rate (HR) and the clinical context (11, 12). However, the DAP-to-HR ratio (DAP/HR) does not account for the NE dose required to achieve the observed arterial pressure. The vascular norepinephrine responsiveness index (VNERi) was recently proposed as an indirect bedside marker of vascular responsiveness and is calculated as DAP divided by the product of NE dose and HR [VNERi = DAP/(NE dose ×  HR)]. In a *post hoc* analysis of the ANDROMEDA-SHOCK database, VNERi showed a stronger association with clinical outcomes than DAP, DAP/HR, and the MAP-to-NE dose ratio (MAP/NE). The association between VNERi and mortality was non-linear, with the lowest estimated mortality risk near a VNERi value of 6.7 (13, 14).

Evidence on VNERi remains limited and largely derived from clinical trial data. Real-world evidence based on routinely collected clinical data can complement trial-derived findings by evaluating feasibility, transportability, and prognostic performance in less selected populations and routine care pathways (16). This is particularly relevant for bedside hemodynamic markers intended for early risk stratification in emergency departments and sepsis-code pathways.

We therefore evaluated the association between VNERi and 28-day in-hospital mortality in a real-world cohort of adults with septic shock treated with NE in the emergency department of a Latin American regional hospital. We also compared the model fit and discrimination of VNERi with those of MAP/NE and explored the risk-stratification performance of both the previously reported value of 6.7 and a cohort-specific exploratory cutoff derived using the Youden index.

## Methods

### Study design and reporting

We conducted a single-center retrospective cohort study using routinely collected clinical data from adults with septic shock treated with norepinephrine in the emergency department between 2023 and 2025. The study was designed to evaluate the association between early hemodynamic indices derived from arterial pressure, heart rate, and norepinephrine dose and 28-day in-hospital mortality.

The manuscript was prepared according to the STROBE statement for observational studies and the RECORD extension for studies using routinely collected health data (17–18).

### Setting and data sources

The study was conducted in the general adult emergency department of Hospital Regional de Duitama, Boyacá, Colombia, a regional referral hospital serving urban and rural populations and managing a mixed medical and surgical caseload. During the study period, the hospital used an institutional sepsis-code pathway for the early recognition and management of patients with suspected or confirmed sepsis. The pathway included clinical screening, early antimicrobial therapy, microbiological sampling, initial fluid resuscitation, hemodynamic assessment, and determination of vasopressor requirements. Sepsis-code pathways have increasingly been evaluated as pragmatic tools for improving process-of-care metrics and outcomes in emergency department sepsis care (19–21).

In all included patients, the institutional sepsis code was activated prospectively and before NE initiation; no patient was included on the basis of retrospective sepsis-code activation. The clinical sequence comprised sepsis-code activation, initial evaluation and assessment of the hemodynamic response to fluids, NE initiation when the MAP target was not achieved, establishment of invasive monitoring, and recording of the index hemodynamic observation.

Data were obtained from the institutional sepsis-code registry and electronic health records. These sources included demographic data, comorbidities, infection source, severity scores, laboratory results, vital signs, vasopressor dose, and hospital outcomes. Because VNERi was proposed after implementation of the registry, it was calculated retrospectively for the present study and was not used to guide clinical decisions.

### Participants

Eligible patients were adults aged 18 years or older with septic shock treated with norepinephrine during their emergency department stay and with available data to calculate the vascular norepinephrine responsiveness index at the predefined index time. Septic shock was defined according to the Sepsis-3 framework as suspected or confirmed infection, vasopressor requirement to maintain MAP ≥65 mmHg, and serum lactate >2 mmol/L despite initial fluid resuscitation (1).

Patients were excluded if they had missing hospital outcome data, missing data required to calculate the hemodynamic indices, pregnancy, terminal illness documented at admission, do-not-resuscitate orders, or therapeutic limitation orders. All eligible patients during the study period were included consecutively. No formal *a priori* sample-size calculation was performed. The study size was determined by the number of consecutive eligible patients identified during the predefined study period.

### Index time and hemodynamic indices

The index time was defined as the first complete protocolized hemodynamic observation recorded after NE initiation and establishment of invasive arterial monitoring during initial stabilization in the emergency department. The index assessment was not conditioned on achievement of the MAP target; therefore, its timing was determined by the protocolized measurement workflow rather than by attainment of a specific arterial pressure value. At this assessment, DAP and MAP were obtained through invasive arterial catheter monitoring in all included patients and were documented together with HR and the current NE infusion rate by designated nursing staff in a single protocolized observation, within a maximum of 10 min after invasive monitoring was established. Thus, the values were not selected separately from continuous monitor trends or different charted time points.

NE was administered as the bitartrate formulation in all included patients, and doses were recorded in µg/kg/min. VNERi was calculated retrospectively as DAP divided by the product of NE dose and HR [VNERi = DAP/(NE dose × HR)] (14). DAP was expressed in mmHg and HR in beats/min. MAP/NE was calculated as MAP divided by NE dose and was expressed per 100-unit increment for analysis and graphical presentation.

For descriptive reporting and multivariable adjustment, serum lactate was defined *a priori* as the first value recorded after NE initiation. This definition aligned the biochemical marker with the hemodynamic exposure window. Therefore, the lactate value summarized in the baseline table may differ from the diagnostic lactate value used to support septic shock classification at sepsis code activation.

### Emergency department management

According to the institutional sepsis-code pathway, initial resuscitation involved crystalloid boluses of 30 mL/kg when clinically indicated, with up to two such boluses administered according to each patient's hemodynamic response (22). The initial MAP target was ≥65 mmHg for all patients, including those with pre-existing chronic hypertension. No higher initial target based on hypertension history was specified for the index assessment. NE was initiated when this target was not achieved after assessment of the hemodynamic response to fluid administration and was the only vasoactive agent administered in the emergency department. The use of vasopressin, dobutamine, other inotropes, and additional vasoactive agents was restricted to subsequent management in the intensive care unit; none of these agents was administered at the index assessment.

The exact patient-level volume administered before NE initiation could not be reliably reconstructed and was therefore not included as a covariate. Daily net fluid balance was not used as a surrogate because it does not specifically quantify pre-vasopressor fluid administration. Mechanical ventilatory support at the index assessment was recorded as a binary variable in the institutional registry and electronic health records. Echocardiography was not routinely available in the emergency department resuscitation area, and no standardized assessment of cardiac function was performed at the index time.

### Outcome

The primary outcome was 28-day in-hospital mortality after emergency department admission. Patients discharged alive before day 28 were considered survivors for the primary analysis. Patients still hospitalized beyond day 28 were classified according to their vital status on day 28.

### Covariates

The prespecified adjustment variables were age, Sequential Organ Failure Assessment (SOFA) score, Charlson comorbidity index, and serum lactate. These variables were selected *a priori* because they represent clinically plausible confounders of the association between hemodynamic status and mortality. Sex, individual comorbidities, and infection source were summarized descriptively but were not included in the primary adjusted models.

### Bias and data quality

Potential sources of bias included selection bias, information bias, misclassification, confounding by indication, and residual confounding. Selection bias may have arisen from the single-center registry-based design and the requirement for availability of the variables needed to calculate the hemodynamic indices. Information bias was possible because the study relied on retrospective extraction of routinely documented clinical data. Although DAP, MAP, HR, and NE dose were recorded together in a single protocolized observation, these variables were not acquired under a dedicated research measurement protocol; therefore, measurement and documentation errors could not be excluded. To mitigate these risks, data cleaning and consistency checks were performed across clinical, hemodynamic, therapeutic, and outcome variables using both the institutional registry and electronic health records.

Misclassification of septic shock or the index assessment remained possible because eligibility and measurement timing were determined from real-world clinical documentation. To reduce this risk, prespecified definitions were applied consistently, and the institutional sepsis code had been activated prospectively before NE initiation in all included patients. Nevertheless, the index assessment reflected routine emergency department stabilization rather than a strictly standardized experimental time point.

Confounding by indication was also relevant because the NE dose was determined by treating clinicians and may have reflected illness severity, treatment timing, fluid status, and local practice patterns. The exact volume of fluid administered before NE initiation and contemporaneous cardiac function could not be reliably incorporated into the analysis, creating additional potential for residual confounding. Accordingly, VNERi was interpreted as a prognostic marker rather than a causal exposure or a direct measure of treatment effect.

## Statistical analysis

Baseline characteristics were summarized for the overall cohort. Categorical variables were reported as counts and percentages, and continuous variables as mean with standard deviation (SD) or median with interquartile range (IQR), according to their distribution.

Logistic regression was used to evaluate the association between each hemodynamic index and 28-day in-hospital mortality. Univariable models were fitted separately for VNERi and MAP/NE. Separate multivariable models were then fitted for each index using the same prespecified adjustment variables: age, SOFA score, Charlson comorbidity index, and serum lactate. VNERi was analyzed per 1-unit increase and MAP/NE per 100-unit increase. Effect estimates were reported as odds ratios with 95% confidence intervals.

Overall model fit was summarized using the model likelihood-ratio *χ*^2^ statistic, Akaike information criterion, and Bayesian information criterion. Akaike and Bayesian information criteria were used descriptively to compare models fitted to the same analytic sample, with lower values indicating better relative fit. Potential non-linearity in the adjusted association between each index and mortality was assessed using restricted cubic splines with four knots placed at the 5th, 35th, 65th, and 95th percentiles of the observed distribution of each index (23). Linear and spline specifications were compared using likelihood-ratio tests, together with Akaike and Bayesian information criteria. Adjusted predicted probabilities of mortality were estimated across clinically relevant values of VNERi.

The discrimination of VNERi and MAP/NE as individual markers was evaluated using receiver operating characteristic (ROC) curves and the corresponding areas under the ROC curve (AUROCs) with 95% confidence intervals. Because both indices were measured in the same patients, their AUROCs were compared using the nonparametric DeLong method for correlated curves (24). Lower values of either index were specified as indicating higher mortality risk.

For exploratory clinical risk stratification, the previously reported VNERi value of 6.7 was evaluated as an externally derived reference point (14). Mortality was compared between patients with VNERi <6.7 and ≥6.7 using risk ratios with 95% confidence intervals. A cohort-specific exploratory cutoff was also identified by maximizing the Youden index, calculated as sensitivity plus specificity minus one (25). Sensitivity, specificity, and the Youden index were summarized for both the previously reported value and the internally derived cutoff, with values below each cutoff classified as indicating higher risk. Because the internal cutoff was derived and evaluated in the same cohort, it was not considered a validated clinical decision threshold.

Missingness was assessed for each variable. The final analytical cohort had complete data for the outcome, hemodynamic indices, and prespecified adjustment variables; therefore, all 238 patients were included in the primary regression and discrimination analyses, and no imputation was performed. All analyses were performed using StataNow version 19.5 (StataCorp LLC, College Station, TX, USA). All tests were two-sided, and a *p* value <0.05 was considered statistically significant.

## Ethical considerations

The study protocol was reviewed and approved by the Research Ethics Committee of Hospital Regional de Duitama, Boyacá, Colombia (approval No. 10112025-3; approved on November 10, 2025), and received institutional endorsement from Universidad Pedagógica y Tecnológica de Colombia (UPTC). The study was conducted in accordance with the ethical principles of the Declaration of Helsinki and applicable Colombian regulations governing health research. Given the retrospective observational design and use of routinely collected clinical data, the Research Ethics Committee waived the requirement for written informed consent for the entire cohort. Patients were not contacted retrospectively. The analytical database was de-identified before analysis, and no individually identifiable information was reported.

## Results

### Study population

During the study period, 215,055 adult emergency department visits were screened. Of these, 29,955 involved an infection-related presentation and 1,867 underwent prospective sepsis-code activation. Among patients with sepsis-code activation, 1,598 did not meet the septic shock criteria and 31 were excluded because of incomplete data required for cohort eligibility. The final analytical cohort comprised 238 patients with septic shock (Figure 1).

**Figure 1:**
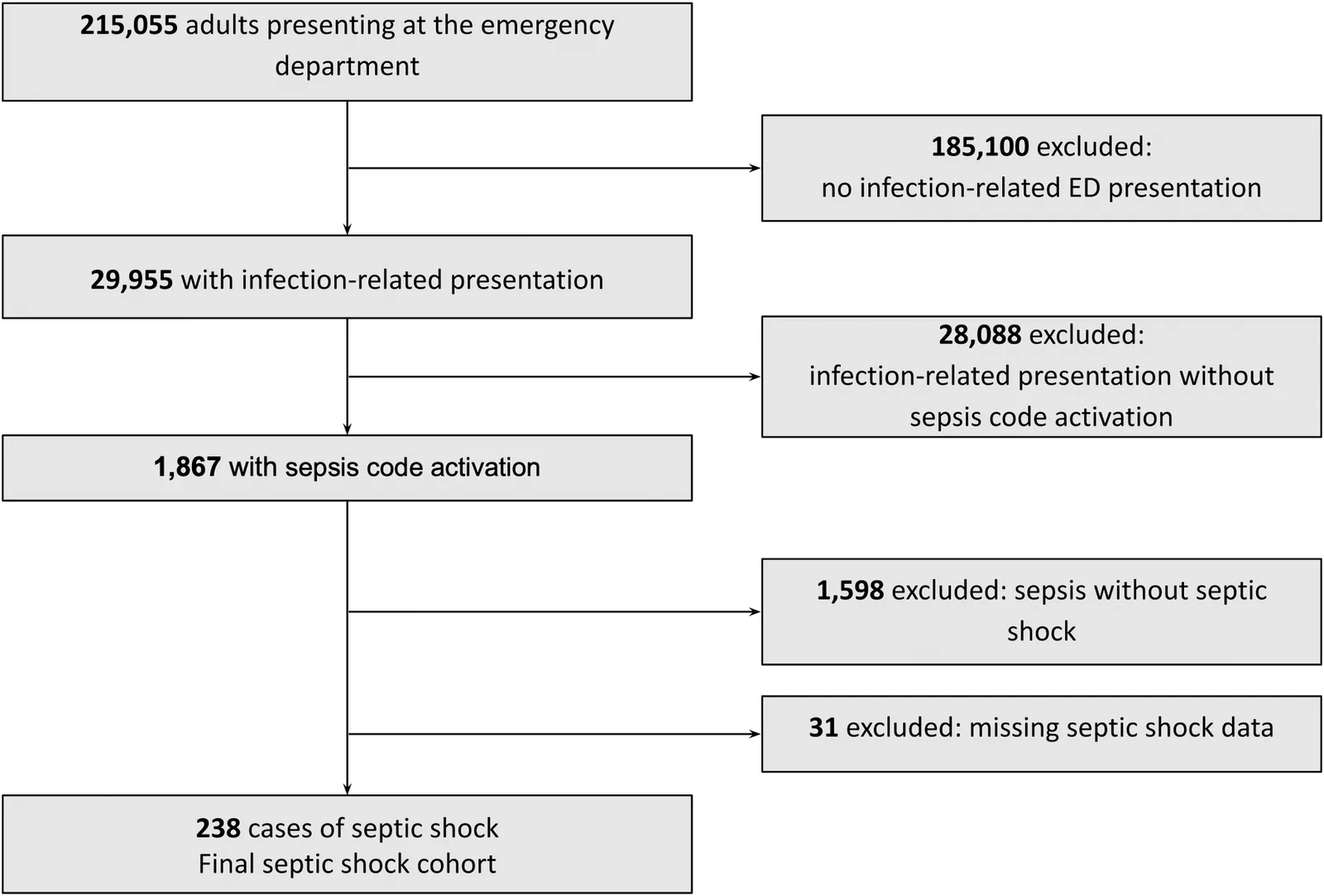
Patient selection flow diagram for septic shock case identification. The flow diagram shows adult emergency department visits, infection-related presentations, sepsis code activations, exclusions, and the final analytical cohort of 238 patients with septic shock.

The analytical cohort included 238 adults with septic shock treated with NE in the emergency department. Median age was 72 years (IQR, 61–81), 126 patients (52.9%) were female, and the median Charlson comorbidity index was 4 (IQR, 3–6). The mean SOFA score was 7.4 (SD, 2.7). The most frequent infection sources were intra-abdominal infection (30.7%), urinary tract infection (29.4%), and pneumonia (20.6%).

All 238 patients underwent invasive arterial pressure monitoring at the index assessment. Median DAP was 61 mmHg (IQR, 54–66), median HR was 89 beats/min (IQR, 75–107), median NE dose was 0.10 µg/kg/min (IQR, 0.05–0.20), and median serum lactate was 3.1 mmol/L (IQR, 1.9–5.8). Median VNERi was 6.2 (IQR, 3.1–12.5). On its original scale, median MAP/NE was 786.7 (IQR, 336.0–1,440.0). NE was the only vasoactive agent administered at the index assessment, and mechanical ventilatory support was recorded in 68 patients (28.6%). Seventy patients (29.4%) experienced the primary outcome of 28-day in-hospital mortality. Baseline characteristics are presented in Table 1. No data were missing for the outcome, hemodynamic indices, or prespecified adjustment variables among the 238 included patients.

**Table 1:** Characteristics of the study cohort.

Characteristic	*N* = 238
Age, median [IQR], years	72 [61–81]
Female sex, *n* (%)	126 (52.9)
Charlson comorbidity index, median [IQR]	4 [3–6]
SOFA score, mean (SD)	7.4 (2.7)
Comorbidities	
Chronic hypertension, *n* (%)	95 (39.9)
Other cardiovascular diseases, *n* (%)	67 (28.2)
Diabetes mellitus, *n* (%)	55 (23.1)
Chronic obstructive pulmonary disease, *n* (%)	52 (21.8)
Chronic kidney disease, *n* (%)	37 (15.5)
Immunosuppression, *n* (%)	26 (10.9)
Other comorbidities, *n* (%)	51 (21.4)
Source of sepsis	
Intra-abdominal infection, *n* (%)	73 (30.7)
Urinary tract infection, *n* (%)	70 (29.4)
Pneumonia, *n* (%)	49 (20.6)
Skin and soft tissue infection, *n* (%)	26 (10.9)
Other source, *n* (%)	11 (4.6)
Unknown source, *n* (%)	9 (3.8)
Hemodynamic variables at the index assessment	
Systolic arterial pressure, median [IQR], mmHg	103 [93–116]
Diastolic arterial pressure, median [IQR], mmHg	61 [54–66]
Mean arterial pressure, median [IQR], mmHg	75 [62.5–82]
Pulse pressure, median [IQR], mmHg	40 [32–51]
Heart rate, median [IQR], beats/min	89 [75–107]
Norepinephrine dose, median [IQR], µg/kg/min	0.1 [0.05–0.2]
VNERi, median [IQR]	6.2 [3.1–12.5]
MAP/NE, median [IQR]	786.7 [336.0–1,440.0]
Serum lactate, median [IQR], mmol/L	3.1 [1.9–5.8]
Support at the index assessment	
Mechanical ventilatory support, *n* (%)	68 (28.6)
Additional vasoactive or inotropic therapy, *n* (%)	0 (0.0)
Outcome	
28-day in-hospital mortality, *n* (%)	70 (29.4)

IQR, interquartile range; SD, standard deviation; SOFA, Sequential Organ Failure Assessment; VNERi, vascular norepinephrine responsiveness index; MAP/NE, mean arterial pressure-to-norepinephrine dose ratio. Serum lactate corresponds to the first lactate value recorded after norepinephrine initiation. Comorbidities were not mutually exclusive. MAP/NE values are presented on their original scale; regression estimates were expressed per 100-unit increase.

### Association between hemodynamic indices and mortality

In univariable analysis, higher VNERi was associated with lower odds of 28-day in-hospital mortality. Each 1-unit increase in VNERi was associated with an odds ratio of 0.84 (95% confidence interval, 0.79–0.90). Higher MAP/NE was also associated with lower odds of mortality when analyzed per 100-unit increase (odds ratio, 0.86; 95% confidence interval, 0.81–0.91).

After adjustment for age, SOFA score, Charlson comorbidity index, and serum lactate in separate multivariable models, both indices remained associated with mortality. Each 1-unit increase in VNERi was associated with lower adjusted odds of death (adjusted odds ratio, 0.85; 95% confidence interval, 0.79–0.91), whereas each 100-unit increase in MAP/NE was associated with an adjusted odds ratio of 0.86 (95% confidence interval, 0.81–0.92). The adjusted VNERi model showed better relative fit than the adjusted MAP/NE model, with a higher model likelihood-ratio *χ*^2^ statistic (53.6 vs. 49.2) and lower Akaike information criterion (246.8 vs. 251.1) and Bayesian information criterion (267.6 vs. 271.9). Regression estimates and model-fit indices are presented in Table 2.

**Table 2:** Univariable and multivariable logistic regression models for 28-day in-hospital mortality.

Model	Index	Effect estimate	LR *χ*^2^	Model *p* value	AIC	BIC
Univariable	VNERi (per 1-unit increase)	OR 0.84 (95% CI, 0.79–0.90)	40.9	*p* < 0.001	251.5	258.4
Univariable	MAP/NE (per 100-unit increase)	OR 0.86 (95% CI, 0.81–0.91)	37.5	*p* < 0.001	254.8	261.7
Multivariable	VNERi (per 1-unit increase)	aOR 0.85 (95% CI, 0.79–0.91)	53.6	*p* < 0.001	246.8	267.6
Multivariable	MAP/NE (per 100-unit increase)	aOR 0.86 (95% CI, 0.81–0.92)	49.2	*p* < 0.001	251.1	271.9

AIC, Akaike information criterion; aOR, adjusted odds ratio; BIC, Bayesian information criterion; CI, confidence interval; LR, likelihood ratio; MAP/NE, mean arterial pressure-to-norepinephrine dose ratio; OR, odds ratio; VNERi, vascular norepinephrine responsiveness index; SOFA, Sequential Organ Failure Assessment. Separate multivariable models were fitted for VNERi and MAP/NE and adjusted for age, SOFA score, Charlson comorbidity index, and serum lactate. All models included 238 patients.

VNERi showed greater discrimination for 28-day in-hospital mortality than MAP/NE (AUROC, 0.737 [95% confidence interval, 0.675–0.799] vs. 0.667 [95% confidence interval, 0.592–0.742]; *p* = 0.012).

### Non-linear association

Restricted cubic spline analysis supported a non-linear adjusted association between VNERi and 28-day in-hospital mortality. Compared with the adjusted linear specification, the spline model provided significantly better fit [likelihood-ratio *χ*^2^(2) = 8.50; *p* = 0.014]. The Akaike information criterion was lower for the spline model than for the linear model (242.3 vs. 246.8), whereas the Bayesian information criterion was slightly lower for the linear model (267.6 vs. 270.1). The adjusted predicted probability of mortality decreased as VNERi increased, with the greatest change observed across lower VNERi values. This association and the previously reported value of 6.7 are shown in Figure 2. In contrast, the spline specification for MAP/NE did not provide a meaningful improvement in relative fit over the linear specification. MAP/NE was therefore retained as a linear comparator.

**Figure 2:**
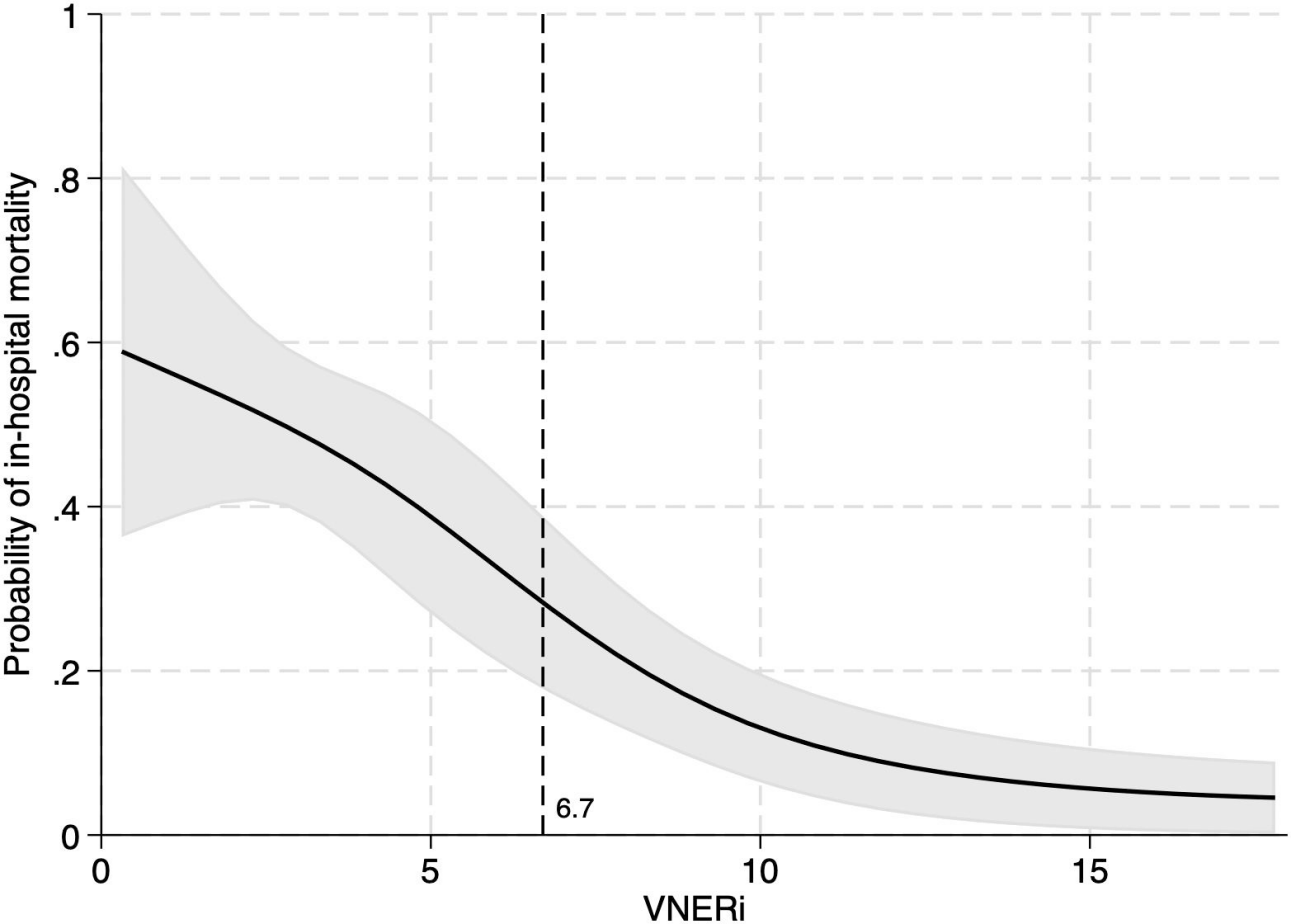
Adjusted probability of 28-day in-hospital mortality according to VNERi. Adjusted predicted probabilities were estimated from a multivariable logistic regression model incorporating a restricted cubic spline for VNERi and adjustment for age, Sequential Organ Failure Assessment score, Charlson comorbidity index, and serum lactate. The vertical dashed line indicates the previously reported VNERi value of 6.7, evaluated in this cohort as an exploratory reference point. The shaded band represents the 95% confidence interval.

### Exploratory VNERi cutoff analyses

At the previously reported VNERi value of 6.7, 125 patients had values <6.7 and 113 had values ≥6.7. Mortality was 48.0% (60/125) in the <6.7 group and 8.8% (10/113) in the ≥6.7 group, corresponding to a crude risk ratio of 5.42 (95% confidence interval, 2.92–10.08; *p* < 0.001). The sensitivity, specificity, and Youden index of this reference value were 85.7%, 61.3%, and 0.470, respectively.

The cohort-specific exploratory cutoff identified by maximizing the Youden index was 7.6, with a sensitivity of 88.6%, specificity of 58.9%, and Youden index of 0.475. Thus, the internally derived cutoff provided only a marginal improvement in Youden performance compared with the previously reported value of 6.7. These exploratory analyses are presented in Table 3. Additional graphical analyses supporting the primary and exploratory findings are presented in Supplementary Figures S1–S10.

**Table 3:** Exploratory risk stratification using the previously reported VNERi value of 6.7 and a cohort-specific cutoff.

Panel A. Mortality stratification at the previously reported VNERi value of 6.7					
VNERi subgroup	N	Death, *n* (%)	Alive, *n* (%)	Crude RR (95% CI)	*p* value
VNERi ≥6.7	113	10 (8.8)	103 (91.2)	Reference	
VNERi <6.7	125	60 (48.0)	65 (52.0)	5.42 (2.92–10.08)	<0.001

Panel B. Classification performance of the evaluated VNERi cutoffs			
VNERi cutoff	Sensitivity, %	Specificity, %	Youden index
Previously reported value: 6.7	85.7	61.3	0.470
Cohort-specific exploratory cutoff: 7.6	88.6	58.9	0.475

CI, confidence interval; RR, risk ratio; VNERi, vascular norepinephrine responsiveness index. The VNERi value of 6.7 was previously reported and was evaluated in this cohort as an externally derived exploratory reference point. The cutoff of 7.6 was derived and evaluated in the same cohort by maximizing the Youden index. Neither value should be interpreted as a validated clinical decision threshold.

## Discussion

In this real-world retrospective cohort of adults with septic shock treated with NE in the emergency department, lower VNERi remained associated with higher 28-day in-hospital mortality after multivariable adjustment. Compared with MAP/NE under the same adjustment structure, VNERi showed modestly better model fit and greater discrimination. The association between VNERi and mortality was non-linear, with the greatest change in predicted mortality observed across lower VNERi values. The previously reported value of 6.7 identified groups with markedly different observed mortality, whereas the cohort-specific cutoff of 7.6 provided only a marginal improvement in Youden performance. Taken together, these findings suggest that VNERi may provide additional prognostic information as a pragmatic early risk-stratification marker, without establishing a role in guiding treatment decisions.

The rationale underlying VNERi is consistent with the concept that septic shock reflects not only hypotension but also heterogeneous vasomotor tone and variable response to catecholamine support. DAP provides indirect information about arterial tone, NE dose reflects the intensity of pharmacological support, and HR provides additional context for interpreting the observed pressure. By integrating these variables, VNERi summarizes the relationship between arterial pressure and vasopressor exposure during early stabilization. A distinct vascular reactivity index incorporating hemodynamic and vasoactive-support variables has also been associated with 28-day mortality in a retrospective cohort of adults with septic shock (15). However, DAP is also influenced by arterial compliance, cardiac function, and other hemodynamic factors, whereas NE dosing depends on clinical decisions, fluid status, and treatment timing. VNERi should therefore be interpreted as a composite hemodynamic and prognostic marker rather than as a direct measurement of vascular tone or catecholamine responsiveness and should always be considered within the broader clinical context (4, 11, 14).

Our findings are consistent with the *post hoc* analysis conducted by Goury et al. using the ANDROMEDA-SHOCK database, in which VNERi was more strongly associated with outcomes than DAP, DAP/HR, and MAP/NE. They described a non-linear, inverted J-shaped association, with the lowest mortality near 6.7 and increasing risk below this value. Our cohort showed a similar low-value pattern, but this does not validate 6.7 as a clinical threshold (14). Methodologically, both studies used NE bitartrate doses expressed in µg/kg/min and invasive arterial monitoring (14, 26). However, Goury et al. assessed hemodynamic variables at randomization within four hours of septic shock diagnosis in 424 patients enrolled in a multicenter ICU trial, whereas our index was calculated from the first protocolized invasive assessment during routine emergency department stabilization in a single-center retrospective cohort. These differences in setting, timing, and data collection limit direct performance comparisons, while our cohort reflects routine emergency department case mix and care pathways (27).

MAP/NE and related MAP/norepinephrine-equivalent dose (MAP/NEQ) indices similarly relate arterial pressure to vasopressor exposure (28). In 19,539 MIMIC-IV ICU records of patients with shock, Yang et al. found that higher MAP/NEQ values within the first 24 h were associated with lower short-term mortality and described an L-shaped relationship (29). Kotani et al. noted that norepinephrine-equivalent dosing can standardize multi-agent vasopressor exposure but remains limited by uncertain conversion ratios and agent-specific hemodynamic effects (30, 31).

In our cohort, NE was the only vasoactive agent administered at the index assessment; therefore, MAP/NE was calculated directly without requiring norepinephrine-equivalent conversion. This avoided uncertainty from conversion ratios but limits generalizability to patients receiving multi-agent vasopressor therapy. VNERi showed greater discrimination for 28-day in-hospital mortality than MAP/NE (AUROC, 0.737 [95% CI, 0.675–0.799] vs. 0.667 [95% CI, 0.592–0.742]; *p* = 0.012). Although statistically significant, this difference was modest and does not establish physiological or clinical superiority or support either index as a standalone prognostic tool. MAP is influenced by both vascular tone and cardiac output, whereas VNERi combines DAP, HR, and NE dose and may capture a different hemodynamic construct (32). Because neither vascular responsiveness nor contemporaneous cardiac function was measured directly, prospective external validation is required before either index can inform treatment decisions.

The real-world nature of this study is both a strength and a limitation. Real-world evidence can clarify how a marker behaves in routine practice rather than under tightly controlled trial conditions (16). Colombian evidence that standardized training improves operational knowledge of Code Sepsis procedures underscores the importance of institutional preparedness, although that study evaluated educational rather than clinical outcomes (21). In our cohort, data from an institutional sepsis-code registry demonstrate that VNERi can be retrospectively reconstructed from variables routinely documented during emergency department care. However, these data were not originally collected to evaluate the index, which should therefore be interpreted as a pragmatic prognostic marker rather than a direct physiological measurement of vascular responsiveness.

Clinically, VNERi may provide additional risk information during early stabilization when interpreted alongside perfusion findings, serum lactate, illness-severity measures, and the overall clinical context. However, a low VNERi should not be interpreted as an indication for any specific intervention. This study did not evaluate VNERi-guided treatment, serial changes in the index, or the optimal interval for reassessment. Therefore, no therapeutic algorithm or reassessment interval can be recommended from these findings. Prospective validation and interventional studies are required before VNERi can be incorporated into clinical decision pathways.

## Strengths and limitations

This study has several strengths. First, it evaluates a recently proposed hemodynamic index in a real-world emergency department cohort, complementing evidence derived from trial data. Second, VNERi was calculated using variables routinely available after establishment of invasive arterial monitoring, without requiring advanced cardiac output monitoring. Third, the analysis used prespecified covariate adjustment, evaluated non-linearity, and directly compared the discrimination of VNERi with that of MAP/NE.

Several limitations should be acknowledged. First, the retrospective single-center design limits causal interpretation and generalizability. Selection bias also remains possible because cohort inclusion depended on prospective sepsis-code activation and the availability of complete eligibility data. Second, although the variables used to calculate VNERi were obtained from a single protocolized observation, they were routinely documented rather than acquired under a dedicated research protocol; therefore, measurement and documentation errors cannot be excluded. Third, VNERi was evaluated at a single assessment during routine stabilization, precluding evaluation of serial changes. Moreover, the exact elapsed time between NE initiation and the index observation could not be reliably reconstructed, limiting temporal standardization.

Fourth, the exact volume of fluid administered before NE initiation could not be reliably reconstructed, and contemporaneous cardiac function was not systematically assessed. Consequently, volume status and resuscitation intensity may have influenced NE requirements and, consequently, VNERi (33), while unmeasured septic myocardial dysfunction remains another potential source of residual confounding. Mechanical ventilatory support was recorded in 68 patients (28.6%) but was not included in the multivariable models. Detailed ventilatory settings, including the applied PEEP, were not available for analysis. Because positive-pressure support may alter venous return, ventricular loading conditions, and cardiac output, unmeasured differences in respiratory support may have influenced the hemodynamic indices and represent an additional source of residual confounding (34).

Fifth, confounding by indication remains possible because NE dosing depends on clinician decisions, illness severity, treatment timing, and local practice. Furthermore, the exclusive use of NE at the index assessment may limit generalizability to patients receiving multi-agent vasopressor therapy.

Sixth, the previously reported value of 6.7 was evaluated as an externally derived exploratory reference, whereas the cutoff of 7.6 was derived and evaluated in the same cohort and is therefore susceptible to optimism; neither should be considered a validated clinical threshold. Finally, this study was not designed to determine whether VNERi-guided treatment improves outcomes.

## Conclusions

In a real-world emergency department cohort of adults with septic shock treated with NE, lower VNERi was independently associated with higher 28-day in-hospital mortality and showed modestly greater discrimination than MAP/NE. These findings support further evaluation of VNERi as a pragmatic early risk-stratification marker. Prospective multicenter validation using standardized measurement timing and external validation of candidate thresholds are required before clinical implementation.

## Data Availability

The data analyzed in this study is subject to the following licenses/restrictions: the dataset contains de-identified clinical data derived from institutional health records and is not publicly available because of ethical, privacy, and institutional data-protection restrictions. Access may be considered upon reasonable request to the corresponding author and is subject to approval by Hospital Regional de Duitama. Requests to access these datasets should be directed to miguel.paiba01@uptc.edu.co.

## Supplementary material

The Supplementary Material for this article can be found online at: https://www.frontiersin.org/articles/10.3389/fmed.2026.1941982/full#supplementary-material

Supplementary Figures S1-S10 containing additional graphical analyses supporting the main and exploratory findings, including covariate-mortality associations, comparisons of linear and non-linear model specifications, VNERi risk-stratification analyses, an exploratory Fagan nomogram, and receiver operating characteristic curves comparing VNERi with MAP/NE
