# Supplementary material for: Vascular norepinephrine responsiveness index for early risk stratification in emergency department septic shock: a real-world cohort study from a Latin American sepsis code registry
Source: Front Med (Lausanne). 2026 Sep 30;13:1941982. doi: 10.3389/fmed.2026.1941982 (PMC13662144; doi:10.3389/fmed.2026.1941982)
Supplement: Supplementary file 1 [file Supplementaryfile1.docx]

**Supplementary material**

**Vascular norepinephrine responsiveness index for early risk stratification in emergency department septic shock: a real-world cohort study from a Latin American sepsis code registry**

Miguel Santiago Paiba Montaña, Juan David Bustos Acosta, Liliana Patricia Acero Galvis, Jonathan Alexander Guezguan Pérez, Yardany Rafael Méndez Fandiño

**Supplementary figures**

This supplementary file contains additional graphical analyses supporting the main manuscript. Tables are not included here because the relevant tabular results are presented in the main manuscript.

Supplementary Figure S1. Association between serum lactate and in-hospital mortality. The graph shows model-estimated predicted probability of in-hospital mortality across lactate values. Shaded areas indicate 95% confidence intervals where applicable.

Supplementary Figure S2. Association between age and in-hospital mortality. The graph shows model-estimated predicted probability of in-hospital mortality across age values. Shaded areas indicate 95% confidence intervals where applicable.

Supplementary Figure S3. Association between Charlson comorbidity index and in-hospital mortality. The graph shows model-estimated predicted probability of in-hospital mortality across Charlson comorbidity index values. Shaded areas indicate 95% confidence intervals where applicable.

Supplementary Figure S4. Association between Sequential Organ Failure Assessment score and in-hospital mortality. The graph shows model-estimated predicted probability of in-hospital mortality across Sequential Organ Failure Assessment score values. Shaded areas indicate 95% confidence intervals where applicable.

Supplementary Figure S5. Linear and non-linear specifications for the vascular norepinephrine responsiveness index. The graph compares the predicted probability of in-hospital mortality using linear and non-linear model specifications.

Supplementary Figure S6. Association between the mean arterial pressure-to-norepinephrine dose ratio and in-hospital mortality. The graph shows model-estimated predicted probability of in-hospital mortality across MAP/NE values expressed per 100-unit increment. Shaded areas indicate 95% confidence intervals where applicable.

Supplementary Figure S7. Linear and non-linear specifications for the mean arterial pressure-to-norepinephrine dose ratio. The graph compares the predicted probability of in-hospital mortality using linear and non-linear model specifications.

Supplementary Figure S8. Distribution of predicted in-hospital mortality by vascular norepinephrine responsiveness index subgroup. Box plots compare predicted mortality distributions for patients with VNERi <6.7 and VNERi ≥6.7.

Supplementary Figure S9. Exploratory Fagan nomogram for the pre-specified vascular norepinephrine responsiveness index threshold of 6.7. This figure is intended only to support clinical interpretation of the exploratory threshold analysis and should not be interpreted as a validated diagnostic test.

**Abbreviations:** MAP/NE, mean arterial pressure-to-norepinephrine dose ratio; SOFA, Sequential Organ Failure Assessment; VNERi, vascular norepinephrine responsiveness index.

Supplementary Figure S10. Receiver operating characteristic curves comparing the discriminative performance of the vascular norepinephrine responsiveness index (VNERi) and the mean arterial pressure-to-norepinephrine dose ratio (MAP/NE) for 28-day in-hospital mortality.
